# Are Classification Criteria for IgG4-RD Now Possible? The Concept of IgG4-Related Disease and Proposal of Comprehensive Diagnostic Criteria in Japan

**DOI:** 10.1155/2012/357071

**Published:** 2012-05-29

**Authors:** Kazuichi Okazaki, Hisanori Umehara

**Affiliations:** ^1^Division of Gastroenterology and Hepatology, The Third Department of Internal Medicine, Kansai Medical University, Shinmachi, Hirakata, Osaka 573-1197, Japan; ^2^Division of Hematology and Immunology, Department of Internal Medicine, Kanazawa Medical University, 1-1 Daigaku, Uchinada-machi, Kahoku-gun, Ishikawa 920-0293, Kanazawa, Japan

## Abstract

Recent studies suggest simultaneous or metachronous lesions in multiorgans characterized by elevated serum levels of IgG4 and abundant infiltration of IgG4-positive plasma cells with various degrees of fibrosis. Two Japanese research committees for IgG4-RD, one from fibrosclerosis (Okazaki team) and the other from lymph proliferation (Umehara team) supported by the “Research Program for Intractable Disease” of the Ministry of Health, Labor, and Welfare of Japan, have agreed with the unified nomenclature as “IgG4-RD” and proposed the comprehensive diagnostic criteria (CDC) for IgG4-RD. Validation of the CDC demonstrated satisfactory sensitivity for the practical use of general physicians and nonspecialists but low sensitivity in the organs to be difficult in taking biopsy specimens such as type1 autoimmune pancreatitis (IgG4-related AIP), compared with IgG4-related sialadenitis/dacryoadenitis (Mikulicz's disease) and IgG4-related kidney disease. Although the diagnostic criteria covering all IgG4-RD are hard to be established, combination with the CDC and organ-specific diagnostic criteria should improve sensitivity.

## 1. Introduction

Recent studies have suggested simultaneous or metachronous lesions in multiorgans characterized by elevated serum levels of IgG4 and abundant infiltration of IgG4-positive plasma cells with various degrees of fibrosis, which lead us to propose the concept of a systemic disease [1, 4, 10, 23, 24]. However, there are many synonyms suggesting a systemic disease, such as IgG4-related autoimmune disease [1], IgG4-related sclerosing disease [4], IgG4-related plasmacytic syndrome (SIPS) [23], IgG4-related multiorgan lymphoproliferative syndrome (IgG4-MOLPS) [10], and systemic IgG4-related disease, all of which may refer to the same conditions [24, 25] (Table 1). To simplify these conditions, members of two Japanese research committees for IgG4-related disease, one from view of fibrosclerosis (Chaired by Prof. Okazaki) [24] and the other from lymph proliferation (Chaired by Professor. Umehara H) [25], both of which are supported by the “Research for Intractable Disease” Program from the Ministry of Health, Labor, and Welfare of Japan, have agreed with unification of different nomenclatures as “IgG4-related disease (IgG4-RD)” and proposed the comprehensive diagnostic criteria (CDC) for IgG4-RD [15]. As it still remains unclear whether pathogenetic mechanisms in each involved organ-are same or not, the term IgG4-RD was appointed as minimally reflecting these conditions to avoid misdiagnosis of malignancy as much as possible.

## 2. The Concept of IgG4-Related Disease

The two Japanese research committees independently analyzed the clinical features and conditions of IgG4-RD and finally resulted in the following consensus with close collaboration [15, 24, 25]. (1) Patients with IgG4-RD show diffuse/focal organ enlargement, with mass-forming or nodular/thickened lesions in various organs, including the central nervous system [26], lachrymal/salivary glands [10, 23], thyroid gland [27, 28], lungs [29], pancreas [30, 31], biliary duct [32], liver [33], gastrointestinal tract [34, 35], kidneys [36], prostate gland [37], retroperitoneum [38], skin [39], lymph nodes [5, 40, 41], and artery [42, 43]. These conditions are quite similar to multifocal idiopathic fibrosclerosis (MIF) [44]. (2) These multiorgan lesions may occur synchronously or metachronously, with the prominent infiltration of lymphocytes and IgG4-positive plasmacytes with fibrosis. (3) IgG4-RD mainly affects middle-aged to elderly men except for IgG4-related dacryoadenitis/sialadenitis. Although clinical symptoms depending on involved organs are relatively mild, some patients develop serious complications such as obstructive jaundice due to hepatic, gallbladder, or pancreatic lesions; hydronephrosis due to retroperitoneal fibrosis; respiratory symptoms due to pulmonary lesions. (4) Steroid treatment is effective in many patient with IgG4-RD. However, prognosis and risk factors of recurrence still remain unclear. (5) Although the infiltration of IgG4-positive cells and increased serum concentrations of IgG4 characteristic of IgG4-RD, the severity of fibrosis is dependent on the individual organs involved. For example, storiform fibrosis and obliterative phlebitis are characteristic of pancreatic, biliary tract, and retroperitoneal lesions but are rarely observed in lachrymal/salivary glands or lymph nodes.

## 3. IgG4-Related Disease (IgG4-RD) as the Comprehensive Nomenclature [24, 25] 

In addition to MIF, there are many synonyms, such as IgG4-related autoimmune disease [1], “IgG4-related sclerosing disease” [4], IgG4-related plasmacytic disease (SIPS) [23], and “IgG4 + sMOLPS” [10], all of which may refer to the same conditions. It has been debated which one is the most appropriate. Storiform fibrosis and obliterative phlebitis are characteristic of biliopancreatic, retroperitoneal, and renal lesions, but rarely observed in lachrymal/salivary glands and lymphnodes [24, 25]. Then, the nomenclature of “IgG4-related sclerosing disease” is mainly based on the fibrous swollen organs, whereas those of “IgG4-SIPS” and “IgG4-MOLPS” are based on lymphoplasmacytic proliferation and swollen lymph nodes without fibrosis [24, 25]. Although most patients have multiorgan lesions synchronously or metachronously, about 10–20% of the patients show a solitary organ involved without confirming other organ involvement [24, 25]. Therefore, it is unclear whether the pathogenetic mechanism is same among individual organs or not. In addition to IgG4-RD, IgG4-associate conditions such as high serum levels of IgG4 or abundant infiltration of IgG4-positive cells were reported in some patients with malignancy; pancreatic [6, 45], biliary [46] and salivary cancer [47], gastrointestinal sarcoma [48], and ocular adnexal lymphoma [49–51]. Therefore, the term “systemic” may lead us to misdiagnosis of other organ lesions showing IgG4-related conditions in cases of malignancy [51]. Based on these findings, the members of Umehara and Okazaki teams have agreed that the term “IgG4-related disease” is appointed as minimally accepting these conditions at this moment.

## 4. Comprehensive Diagnostic Criteria for IgG4-RD [15, 24, 25]

 The patients with IgG-4-related disease show organ enlargement or nodular/hyperplastic lesions in organs in the entire body, synchronously or metachronously, due to the prominent infiltration and fibrosis of lymphocytes and plasmacytes; however, the causes of the disease are still not clear. The organs known to be affected include the central nervous system, lacrimal/salivary glands, thyroid gland, lungs, pancreas, biliary duct, liver, gastrointestinal tracts, kidneys, prostate gland, retroperitoneum, skin, arteries, and lymph nodes. Although it remains unclear whether this disease is the same as multifocal fibrosclerosis, that is a possibility. Clinical symptoms vary depending on the organ in which the lesions are located, which suggests that it is hard to establish criteria covering all patients with IgG4-RD. Therefore, specific diagnostic criteria are required for each involved organ such IgG4-related Mikulicz's disease (IgG4-related dacryoadenitis/sialadenitis [12] (Table 2), type 1 AIP (IgG4-related pancreatitis) [13] (Table 3), and IgG4-related kidney disease [14, 41] (Table 4). However, these organ-specific criteria do not cover other organs or are not familiar to general clinicians and specialists. Moreover, to avoid misdiagnosis of malignancy, all physicians have to know this emerging disease entity and can make a diagnosis of IgG4-RD. Therefore, the CDC for IgG4-RD, containing three major criteria (clinical, hematological and histopathological examinations), have been proposed for practical use of general physicians and nonspecialist [15] (Table 5). Although sensitivity of the CDC for definitive IgG4-RD is low in the organs to be difficult in taking biopsy specimens, it can detect possible cases of IgG4-RD. In the probable or possible cases, organ specific criteria should be used concurrently.

**(1) Clinical Examination**

**Figure 1:**
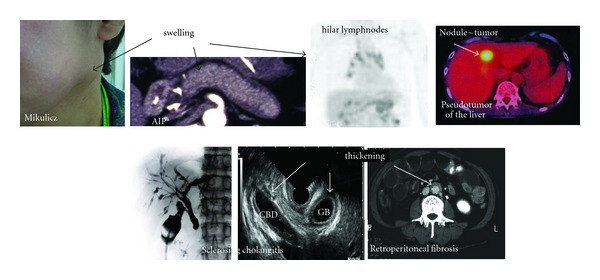
Clinical findings of IgG4-related disease. Physical examinations and imaging on US/CT/MRI can show the characteristic diffuse/localized swelling, masses, or thickness in single or multiple organs.

Physical examinations and imaging on US/CT/MRI can show the characteristic diffuse/localized swelling, masses, or thickness in single or multiple organs (Figure 1).

**(2) Immunological Examination**

**(a) Increase of Serum Levels of IgG4**

The cutoff value for serum IgG4 concentration, 135 mg/dL, was based on receiver operating characteristic (ROC) curves, and its validity was confirmed in patients with autoimmune pancreatitis [7] (Table 6). In patients with single-organ involvement and serum IgG4 concentration less than 135 mg/dL, the IgG4/IgG ratio may be helpful in making a diagnosis.

However, elevated IgG4 may be also observed in other diseases (e.g., atopic dermatitis, pemphigus, asthma, and multicentric Castleman's disease), especially in about 10% of malignancy, which suggests that high serum IgG4 is not necessarily specific marker of IgG4-RD [6]. Although a high cut-off value with >270 mg/dL of IgG4 increases specificity but decreased sensitivity of IgG4-RD differing from pancreatic cancer [45]. Therefore, at present, the significance of elevated IgG4 in the pathogenesis/pathophysiology of IgG4-RD still remains unknown.

**(b) Other Immunological Markers**

In addition to increased serum levels of IgG4, high serum levels of polyclonal *γ*-globulin, IgG, and IgE are often, and hypocomplementemia may occur [52]. As these markers are less sensitive for IgG4-RD, they are not included as a diagnostic criterion.

**(3) Histopathologic Examination**

Although tissue biopsies are difficult to obtain from some organs, including the pancreas, retroperitoneum and ocular cavity, histopathological examination is important.

**(a) Marked Lymphocyte and Plasmacyte Infiltration and Fibrosis.**

Storiform or swirling fibrosis or obliterative phlebitis is Characteristic of IgG4-RD and may be important in its diagnosis.

**(b) Infiltration of IgG4-Positive Plasma Cells**

IgG4/IgG-positive cells more than 40% [53] or 50% [12] have been reported in lymphnodes of the patients with IgG4-RD. On the other hand, more than 10 IgG4-positive plasma cells are recommended that in diagnosis of type 1 AIP [13]. Based on these findings, the CDC for IgG4-RD recommend both the ratio of IgG4/IgG-positive cells >40% and infiltration of >10 IgG4-positive plasma cells/HPF for the definitive diagnosis [15]. Eosinophilic infiltration is often observed along with infiltration of IgG4-positive cells. It is noted that reactive infiltration of IgG4-positive cells and fibrosis may be observed in various diseases and clinical conditions, such as rheumatoid synovitis, inflammatory oral and skin lesions, and around cancer. However, it is noted that some additional immune-mediated conditions with increased serum interleukin-6 (IL-6) such as multicentric Castleman's disease may show elevated serum IgG4 and/or IgG4+/IgG+ plasma cell ratios >40%.

**(4) Prohibition of Facile Steroid Treatment in the CDC for IgG4-RD**

Patients with malignant lymphoma or paraneoplastic lesions can sometimes be improved by steroid administration. Therefore, steroid trials should be strictly avoided. Efforts should be made to collect tissue samples for diagnosis. However, patients having disease in organs difficult to biopsy, such as the pancreas, retroperitoneum, and pituitary, and respond to steroids may possibly have IgG4-RD. In accordance with the guidelines for treatment of autoimmune pancreatitis, patients should be started on 0.5-0.6 mg/kg/day/prednisolone. If patients do not respond to the initial steroid therapy, the diagnosis should be reviewed again.

**(5) Diseases to be Excluded or Differentiated**

**(a) Malignancies (e.g., Cancer, Lymphoma)**

In cases of malignancy in the involved organs, it is essential to determine whether malignant cells are present histopathologically.

**(b) Similar Diseases**

Other similar benign diseases including Sjögren's syndrome, primary sclerosing cholangitis, multicentric Castleman's disease, idiopathic retroperitoneal fibrosis, Wegener's granulomatosis, sarcoidosis, and Churg-Strauss syndrome should be differentially diagnosed using the diagnostic criteria for each disease. It is noted that multicentric Castleman's disease, one of hyper IL-6 syndromes should be excluded from IgG4-RD, even if the CDC for IgG4-RD are fulfilled.

## 5. Sensitivity and Specificity of the CDC Criteria and Diagnostic Algorithm for IgG4-RD

The sensitivity of CDC for definitive/probable IgG4-RD is satisfactory in IgG4-related MD [12] and IgG4-related KD [14], but not in type 1 AIP [6, 13]. The major reason of low sensitivity in type 1 AIP is that enough biopsy samples of the pancreas are not easily obtained in most of these patients. In addition, endoscopic ultrasonography (EUS), guide fine needle aspiration (FNA), is available in a few of institutes in Japan, for examples only 16 of 226 (7%) board member institutes in Kink district of Japan Gastroenterological Endoscopy Society (JGES). On the other hand, the sensitivity of the CDC for possible IgG4-RD is satisfactory in type 1 AIP (Table 7). In contrast, patients with type 1 AIP could not be diagnosed by the comprehensive diagnostic criteria (0%) for definite, because biopsies could not be obtained from most of these patients. Therefore, combination of the CDC and organ-specific criteria should increase the sensitivity of diagnosis, even in the possible cases of IgG4-RD.

**Figure 2:**
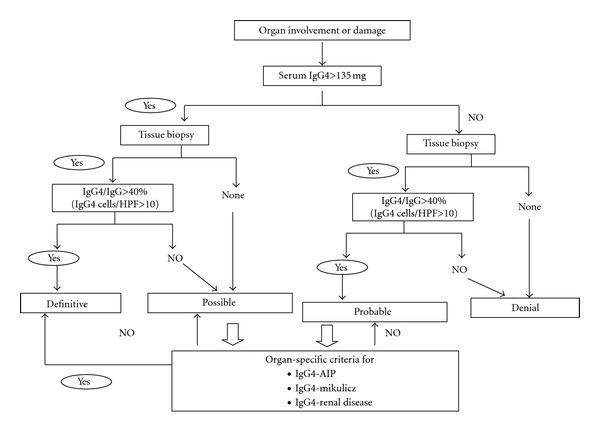
Diagnostic algorithm for IgG4-RD in Japan.

Based on these findings, a diagnostic algorithm for IgG4-RD in combination with the CDC and other organ-specific criteria has been proposed, although they have a limitation to the utility of the criteria proposed [15] (Figure 2). In patients with (a) organ enlargement, mass or nodular lesions, or organ dysfunction, performing of both (b) measurement of serum IgG4 and (c) tissue biopsy is recommended. In the cases with >135 mg/dL of IgG4, diagnostic histopathological findings of >10 IgG4 cells/HPF and an IgG4/IgG cell ratio >40 can diagnose them as definitive AIP. In possible or probable cases fulfilling criterion (a) with (b), or (c), organ-specific criteria for each disease should be applied. It is important to differentiate IgG4-RD from malignant tumors of each organ (e.g., cancer, lymphoma) and similar diseases (e.g., Sjögren's syndrome, primary sclerosing cholangitis, Castleman's disease, secondary retroperitoneal fibrosis, Wegener's granulomatosis, sarcoidosis, and Churg-Strauss syndrome) by additional histopathological examination. Future studies including other organ diseases similar to IgG4-RD are needed to establish the diagnostic efficacy of CDC.

## 6. Conclusion

“All Japan Research Team for IgG4-RD” unified the nomenclatures as “IgG4-related disease (IgG4-RD)” and proposed the comprehensive diagnostic criteria (CDC) for IgG4-RD. The CDC for IgG4-RD was made for the practical use and for general physicians to differentiate IgG4-RD from malignancy or similar diseases as much as possible. Although sensitivity of the CDC for definitive IgG4-RD is low in the organs to be difficult in taking biopsy specimens, it can detect possible cases of IgG4-RD. In the probable or possible cases, organ-specific criteria should be used concurrently.

## Figures and Tables

**Table 1:** Nomenclatures of IgG4-related conditions.

Nomenclature	Authors	(year)
IgG4-related autoimmune disease	Kamisawa et al. [1]	(2003)
IgG4-associated multifocal systemic fibrosis	van der Vliet and Perenboom [2]	(2004)
IgG4-related systemic disease	Kamisawa et al. [3]	(2004)
IgG4-related sclerosing disease	Kamisawa et al. [4–7]	(2006)
Hyper-IgG4 disease	Neild et al. [8]	(2006)
IgG4-related disease	Zen et al. [9]	(2007)
Systemic IgG4 plasmacytic syndrome (SIPS)	Masaki et al. [10]	(2009)
IgG4-related multiorgan Lymphoproliferative syndrome (IgG4-MOLPS)	Masaki et al. [10]	(2009)
IgG4-associated disease	Geyer et al. [11]	(2010)

**Table 2:** Diagnostic criteria for IgG4+ Mikulicz's disease [12] (approved by the Japanese Society for Sjögren's Syndrome, 2008).

(1) Symmetrical swelling of at least 2 pairs of lachrymal, parotid, and submandibular glands continuing for more than 3 months,	
(2) elevated serum IgG4 (>135 mg/dL),	
or	
(3) histopathological features including lymphocyte and IgG4+ plasma cell infiltration (IgG4+ plasma cells/IgG+ plasma cells > 50%) with typical tissue fibrosis or sclerosis.	
Differential diagnosis is necessary from other disorders, including sarcoidosis, Castleman's disease, Wegener's granulomatosis, lymphoma, and cancer. Although the diagnostic criteria for Sjögren's syndrome (SS) may also include some patients with IgG4+ Mikulicz's disease, the clinicopathological conditions of patients with typical SS and IgG4+ Mikulicz's disease are different.	

**Table 3:** International Consensus Diagnostic Criteria (ICDC) for autoimmune pancreatitis [13].

Diagnosis	Primary basic for diagnosis	Imaging Evidence	Collateral evidence
Definitive type 1 AIP	Histology	Typical/indeterminate	Histologically confirmed LPSP (level 1 H)
	Imaging	Typical	Any non-D level 1/level 2
		Indeterminate	Two or more from level 1 (+level 2 D*)
	Response to steroid	Indeterminate	Level 1 S/OOI + Rt or level 1 D + level 2 S/OOI/H + Rt

Probable type 1 AlP		Indeterminate	Level 2 S/OOI/H + Rt

*Level 2 D is counted as level 1 in this setting.			

	Criterion	Level 1	Level 2

P	Parenchymal imaging	Typical: diffuse enlargement with delayed enhancement (sometimes associated with rim-like enhancement)	Indeterminate (including atypical^†^): segmental/focal enlargement with delayed enhancement

D	Ductal imaging (ERP)	Long (>1/3 length of the main pancreatic duct) or multiple strictures without marked upstream dilatation	Segmental/focal narrowing without marked upstream dilatation (duct size, <5 mm)

S	Serology	IgG4, >2× upper limit of normal value a or b	IgG4, 1-2× upper limit of normal value a or b
OOI	Other organ involvement		
		(a) Histology of extrapancreatic organs:	(a) Histology of extrapancreatic organs including endoscopic biopsies of bile duct^‡^:
		any three of the following:	both of the following:
		(1) marked lymphoplasmacytic infiltration with fibrosis and without granulocytic infiltration;	(1) marked lymphoplasmacytic infiltration without granulocytic infiltration;
		(2) storiform fibrosis;	(2) abundant (>10 cells/HPF) IgG4-positive cells.
		(3) obliterative phlebitis;	
		(4) abundant (>10 cells/HPF) IgG4-positive cells.	
		(b) Typical radiological evidence	(b) Physical or radiological evidence:
		at least one of the following:	at least one of the following:
		(1) segmental/multiple proximal (hilar/intrahepatic) or proximal and distal bile duct stricture;	(1) symmetrically enlarged salivary/lachrymal glands;
		(2) retroperitoneal fibrosis;	(2) radiological evidence of renal involvement described in association with AIP.

H	Histology of the pancreas	LPSP (core biopsy/resection):	LPSP (core biopsy):
		at least 3 of the following:	any 2 of the following:
		(1) periductal lymphoplasmacytic infiltrate without granulocytic infiltration;	(1) periductal lymphoplasmacytic infiltrate without granulocytic infiltration;
		(2) obliterative phlebitis;	(2) obliterative phlebitis;
		(3) storiform fibrosis;	(3) storiform fibrosis;
		(4) abundant (>10 cells HPF) IgG4-positive cells.	(4) abundant (>10 cells/HPF) IgG4-positive cells.

		Diagnostic steroid trial	
Response to steroid (Rt)*	Rapid (≤2 wk) radiologically demonstrable resolution or marked improvement in pancreatic/extrapancreatic manifestations		

**Table 4:** Diagnostic criteria for IgG4-related kidney disease [14].

(1) Presence of some kidney damage, as manifested by abnormal urinalysis or urine marker(s) or decreased kidney function with either elevated serum IgG or IgE or hypocomplementemia	
(2) Abnormal renal radiologic findings:	
(a) multiple low-density lesions on enhanced computed tomography;	
(b) diffuse kidney enlargement;	
(c) hypovascular solitary mass in the kidney;	
(d) hypertrophic lesion of the renal pelvic wall without irregularities of the renal pelvic surface.	
(3) Elevated serum IgG4 level (>135 mg/dL)	
(4) Histological findings in the kidney:	
(a) dense lymphoplasmacytic infiltration by >10 IgG4-positive plasma cells/high power field (HPF) and/or IgG4+/IgG+ positive plasma cells > 40%;	
(b) characteristic (sclero-) fibrosis surrounding nests of lymphocytes and/or plasma cells;	
(5) Histological findings in extrarenal organ(s):	
dense lymphoplasmacytic infiltration by >10 IgG4-positive plasma cells/HPF and/or IgG4/IgG-positive plasma cells > 40%	
Definite: (1) + (3) + (4) (a), (b)	
(2) + (3) + (4) (a), (b)	
(2) + (3) + (5)	
(1) + (3) + (4) (a) + (5)	
Probable: (1) + (4) (a), (b)	
(2) + (4) (a), (b)	
(2) + (5)	
(3) + (4) (a), (b)	
Possible: (1) + (3)	
(2) + (3)	
(1) + (4) (a)	
(2) + (4) (a)	
Appendix:	
(1) Clinically and histologically, the following diseases should be excluded:	
Wegener's granulomatosis, Churg-Strauss syndrome, and extramedullary plasmacytoma.	
(2) Radiologically, the following diseases should be excluded:	
Malignant lymphoma, urinary tract carcinomas, renal infarction, and pyelonephritis.	
(Rarely, Wegener's granulomatosis, sarcoidosisand metastatic carcinoma)	

**Table 5:** Comprehensive diagnostic criteria for IgG4-related disease (IgG4-RD), 2011 [15].

*[Concept]*	
IgG4-related disease (IgG4-RD) shows organ enlargement or nodular/hyperplastic lesions in various organs concurrently or metachronously, due to marked infiltration of lymphocytes and IgG4-positive plasma cells, as well as fibrosis of unknown etiology. IgG4-RD affects various organs, including the pancreas, bile duct, lacrimal gland, salivary gland, central nervous system, thyroid, lung, liver, gastrointestinal tract, kidney, prostate, retroperitoneum, arteries, lymph nodes, skin, and breast. Although many patients with IgG4-RD have lesions in several organs, either synchronously or metachronously, others show involvement of a single organ. Clinical symptoms vary depending on the affected organ, and some patients may experience serious complications, such as obstruction or compression symptoms due to organomegaly or hypertrophy and organ dysfunction caused by cellular infiltration or fibrosis. Steroid therapy is often effective.	

*[Comprehensive clinical diagnostic criteria for IgG4-RD,* *2011] *	
(1) Clinical examination shows characteristic diffuse/localized swelling or masses in single or multiple organs.	
(2) Hematological examination shows elevated serum IgG4 concentrations (≥135 mg/dL).	
(3) Histopathologic examination shows;	
(1) marked lymphocyte and plasmacyte infiltration and fibrosis	
(2) infiltration of IgG4-positive plasma cells: ratio of IgG4/IgG positive cells > 40% and > 10 IgG4-positive plasma cells/HPF.	
Definite: (1) + (2) + (3), Probable: (1) + (3), Possible: (1) + (2)	
However, it is important to differentiate IgG4-RD from malignant tumors of each organ (e.g. cancer, lymphoma) and similar diseases (e.g. Sjögren's syndrome, primary sclerosing cholangitis, Castleman's disease, secondary retroperitoneal fibrosis, Wegener's granulomatosis, sarcoidosis, and Churg-Strauss syndrome) by additional histopathological examination. Even when patients cannot be diagnosed using the CCD criteria, they may be diagnosed using organ-specific diagnostic criteria for IgG4RD.	

**Table 6:** Sensitivity and specificity of serum levels of IgG4 in patients with type 1 AIP.

	Cut-off	Sensitivity		Specificity	
	mg/dL	*n *	Median*/*(range)	*n*	(vs cancer)
Japan	135				
Okazaki et al. [16]		71	80% 410 (3–3670)	101	98%
Okazaki et al. [17]		52	73% 505 (43–1540)		NS
Kawa et al. [18]		64	92% 618 (8–2855)	80	98%
Korea	135				
Choi et al. [19]		30	73% 473 (10–1764)	76	99%
USA	140				
Ghazale et al. [20]		45	76% 550 (16–2890)	135	90%
Raina et al. [21]		26	44% (8–825)	NS	
Italy	135				
	(focal)	55	66% 267		NS
Frulloni et al. [22]	(diffuse)	32	27% 78		

**Table 7:** Validation of a combination of CDC and organ-specific criteria for type 1 AIP.

Compared with pancreas cancer, the sensitivity of comprehensive criteria for definite/probable AIP was 0%, but 78% for possible AIP, and specificity was 100% in any groups. Although it is hard to take an enough size of specimen in diagnosis of AIP malignancy can be usually denied by EUS-FNA. Therefore, the CDC are enough for detecting possible AIP, but not for definite/probable AIP.				
AIP (*n* = 60) PaCa (*n* = 17) Total (*n* = 77)	JPS 2006	ICDC for type 1 AIP	CDC for IgG4-RD	

Diagnosis of AIP	Definite AIP	Definite/probable AIP	Definite/probable AIP	Possible

sensitivity	70%	97%	0%	78%
specificity	100%	100%	100%	100%
PPV	100%	100%	0%	100%
NPV	49%	8%	100%	57%
accuracy	77%	95%	22%	83%

PaCa: pancreas cancer, PPV: positive predictive value, NPV: negative predictive value.
